# Prevalence and Correlates of Sinonasal Symptom Burden in Young Adult Males: A Cross-Sectional Population-Based Study (PrENT Study)

**DOI:** 10.3390/life16081318

**Published:** 2026-08-12

**Authors:** Emanuel Maitz, Reinhard Domanyi, Gerold Schwantzer, Gerald Sendlhofer, Alexandros Andrianakis, Dietmar Thurnher, Thomas Weiland

**Affiliations:** 1 Department of Otorhinolaryngology, Head and Neck Surgery, Medical University of Graz, 8010 Graz, Austria; rkd@outlook.at (R.D.); alexandros.andrianakis@medunigraz.at (A.A.); dietmar.thurnher@medunigraz.at (D.T.); thomas.weiland@medunigraz.at (T.W.); 2 Military Hospital Graz, 8052 Graz, Austria; 3 Institute for Medical Informatics, Statistics and Documentation, Medical University of Graz, 8010 Graz, Austria; gerold.schwantzer@medunigraz.at; 4 Division of Plastic, Aesthetic and Reconstructive Surgery, Medical University of Graz, 8010 Graz, Austria; gerald.sendlhofer@medunigraz.at

**Keywords:** chronic rhinosinusitis, SNOT-22, unified airway, C-reactive protein, eosinophils, spirometry, population-based study, young adult males

## Abstract

Objectives: Chronic rhinosinusitis (CRS) is a prevalent chronic inflammatory upper airway disease. Despite a higher radiological disease burden in males, clinical CRS diagnoses are disproportionately registered in females, suggesting systematic underdiagnosis in young men. The 22-item Sinonasal Outcome Test (SNOT-22) represents the reference patient-reported outcome measure (PROM) for sinonasal symptom burden. This study aimed to determine the prevalence of sinonasal symptom burden and to identify associated clinical, laboratory, and lifestyle parameters in a large cohort of young male adults. Methods: In this prospective cross-sectional study, 500 young male adults underwent, as part of the conscription examination, standardized assessment including the SNOT-22, spirometry (FEV_1_%), whole blood count (eosinophils), and serum CRP. SNOT-22 scores were categorized as none (0–20), moderate (21–50), and severe (>50). Associations were analyzed using Spearman correlation, Mann–Whitney U, and Chi^2^ tests. Results: The mean SNOT-22 score was 18.8 (SD 15.2; range 0–73). A total of 38.2% of participants scored ≥21, indicating at least moderate sinonasal symptom burden. SNOT-22 correlated significantly with CRP (r = 0.117, *p* = 0.009) and inversely with relative FEV_1_% (r = −0.103, *p* = 0.022). Eosinophils showed a numeric gradient across SNOT-22 categories (3.08% vs. 3.31% vs. 3.56%) without reaching significance. Conclusions: More than one third of young male adults reported significant self-reported sinonasal symptom burden. Symptom severity was associated with systemic inflammatory markers and reduced pulmonary function, supporting the unified airway concept. These findings support the utility of SNOT-22 as a low-threshold assessment tool for self-reported sinonasal symptom burden in preventive health examinations.

## 1. Introduction

Chronic rhinosinusitis (CRS) affects approximately 10.9% of the European adult population according to the GA^2^LEN survey (n = 57,128), with prevalences ranging from 6.9% in Finland to 27.1% in Portugal [1]. CRS constitutes one of the most common chronic inflammatory upper airway diseases and imposes a substantial socioeconomic burden, with indirect costs exceeding USD 20 billion per annum in the United States [2].

Despite this high burden, a systematic diagnostic gap exists in young males. Computed tomography-based studies identify CRS radiological changes in 8.7–16.1% of male subjects [3], yet administrative databases predominantly register female CRS patients (67.7% in the Olmsted County cohort) [4]. This discrepancy likely reflects lower healthcare-seeking behaviour among young men and possible symptom habituation [5].

The SNOT-22 is the internationally validated reference PROM for CRS, demonstrating excellent psychometric properties [6,7]. Its German-language version has been validated, confirming full equivalence with the original [8].

Beyond symptom assessment, CRS is mechanistically linked to systemic inflammation and lower airway disease through the unified airway concept [9]. Eosinophilic inflammation characterises the type-2 endotype of CRS and predicts disease severity, polyp recurrence, and biological therapy response [10]. Concurrently, reduced FEV_1_% has been observed in CRS patients, reflecting shared mucosal inflammation across the upper and lower airways [11].

Population-based studies examining sinonasal symptom burden alongside objective biomarkers specifically in young male adults are lacking. The PrENT Study was designed to address this gap by applying a comprehensive multiparameter assessment in a large unselected cohort. The present analysis addresses: (1) What is the prevalence of sinonasal symptom burden in young male adults? (2) Which clinical, laboratory, and lifestyle parameters are associated with SNOT-22 scores?

## 2. Materials and Methods

### 2.1. Study Design and Population

The data presented here are part of the PrENT Study (Prevalence of ENT Diseases, NCT06105346), a prospective, single-centre cross-sectional cohort study conducted at the Austrian Military Conscription Commission in Graz, Austria (May 2024–June 2025), under the auspices of the Department of Otorhinolaryngology, Head and Neck Surgery, Medical University of Graz. All male Austrian conscripts aged 17 to 19 years are legally required to attend; during the study period, all were invited to participate and those consenting additionally completed an ENT symptom questionnaire.

The study was conducted in accordance with the Declaration of Helsinki and received ethics approval from the Medical University of Graz (approval no. 36-090ex23/24; 8 March 2024). Written informed consent was obtained from all participants. No participant presented with acute illness; data on prior CRS treatments, allergy status, and seasonal influences were not collected.

### 2.2. SNOT-22 Assessment

Sinonasal symptom burden was assessed using the validated German-language SNOT-22 [8]. Each of the 22 items is rated on a 6-point Likert scale (0 = no problem, 5 = worst possible), yielding a total score of 0–110. Scores were categorised as none (0–20), moderate (21–50), and severe (>50) [6,12].

### 2.3. Spirometry

Spirometry was performed according to ERS/ATS standards. The parameters recorded were: FEV_1_ (l), vital capacity (VC, l), and relative FEV_1_ (FEV_1_/VC, %). Because relative FEV_1_ is expressed as a ratio to the individual’s own VC, values > 100% are not physiologically possible and indicate a measurement or transcription artefact rather than a true finding, unlike FEV_1_% predicted, which can legitimately exceed 100% in young, healthy cohorts. Such cases (n = 10) were therefore excluded, yielding a valid n = 490. For the same reason, the threshold applied here (relative FEV_1_ < 75%) is a pragmatic screening cut-off for this cohort and is not equivalent to a formal diagnosis of ventilatory obstruction, which per current standards requires FEV_1_/FVC below the lower limit of normal (z-score < −1.645) or <0.7; this distinction is addressed further as a limitation in Section 4.4.

### 2.4. Laboratory and Additional Parameters

Venous blood samples were collected under standardised conditions. The parameters analysed included CRP (mg/dL; measured using a standard, non-high-sensitivity clinical chemistry assay), automated differential blood count (relative values: eosinophils%, neutrophils%, lymphocytes%), leucocytes, erythrocytes, total cholesterol, and triglycerides. BMI was calculated from measured height and weight; waist circumference was measured at the umbilical level. Tobacco use was assessed by self-report (smoker: yes/no).

### 2.5. Statistical Analysis

Statistical analyses were performed by the Institute for Medical Informatics, Statistics and Documentation, Graz, using IBM SPSS Statistics v29.0. Continuous data are presented as mean (SD) and median (IQR). Associations between continuous variables were assessed by Spearman rank correlation (r). Group comparisons of continuous variables were performed using the Mann–Whitney U test (two groups) or the Kruskal–Wallis test (three SNOT-22 categories). Categorical associations were examined by the Chi-square test (exact *p*-values). No correction for multiple comparisons (e.g., Bonferroni) was applied, as the tested associations were pre-specified and hypothesis-driven based on the unified airway concept and established CRS-related biomarkers rather than an unrestricted exploratory screen; all reported *p*-values are therefore nominal and associations should be interpreted as hypothesis-generating rather than confirmatory. Significance was set at *p* < 0.05 (two-tailed).

## 3. Results

### 3.1. Study Population and SNOT-22 Distribution

**Figure 1:**
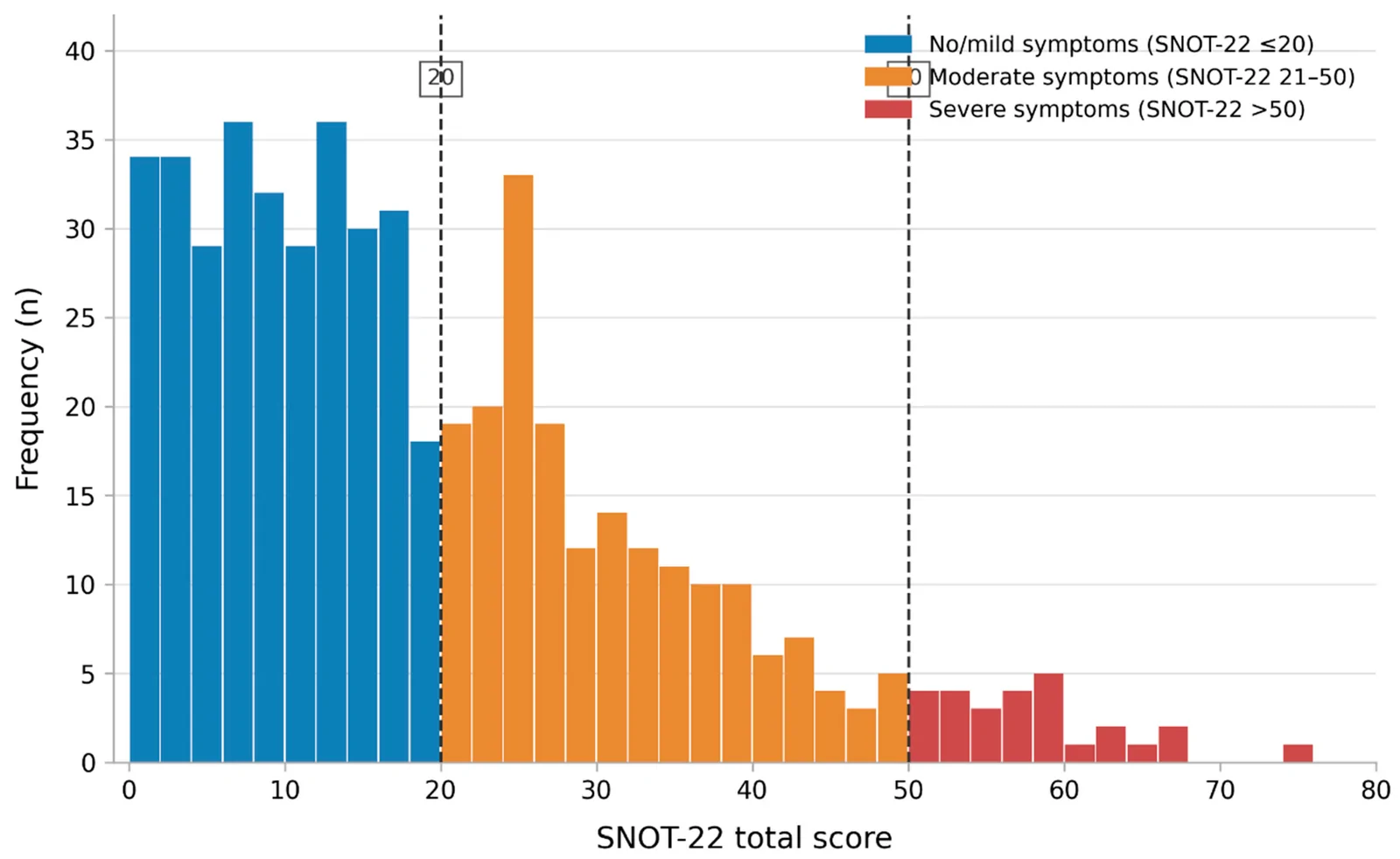
Distribution of SNOT-22 total scores in young male adults (n = 500).

Five hundred young male adults were included. The mean SNOT-22 total score was 18.8 (SD 15.2; median 15.0; IQR 7.0–27.0; range 0–73). The distribution of SNOT-22 categories is shown in Table 1. A total of 191 participants (38.2%) met the criteria for at least moderate sinonasal symptom burden (SNOT-22 ≥ 21): 168 (33.6%) moderate and 23 (4.6%) severe (Figure 1). Item-level analysis revealed the highest mean impairment for fatigue on awakening (item 2.16), nasal blockage (2.2), and need to blow nose (2.1).

### 3.2. SNOT-22 and Systemic Inflammatory Markers

CRP showed a significant positive correlation with SNOT-22 total score (r = 0.117, *p* = 0.009; Table 2). Descriptively, median CRP was 0.00 mg/dL in the no-symptom group, rising to 0.10 mg/dL in both symptomatic groups. Mean CRP was highest in the moderate group (0.13 mg/dL vs. 0.11 in none and 0.12 in severe), consistent with a non-linear pattern. Erythrocyte count also showed a small but significant positive correlation with SNOT-22 (r = 0.091, *p* = 0.042). Triglycerides showed a gradient across categories (mean 87.3 vs. 95.0 vs. 110.2 mg/dL; r = 0.084, *p* = 0.059).

Relative eosinophil values showed a numeric gradient across SNOT-22 categories: mean 3.08% (none) vs. 3.31% (moderate) vs. 3.56% (severe), *p* = 0.159 for correlation. Median eosinophils followed the same direction (2.50 vs. 2.70 vs. 3.10%) without reaching significance.

### 3.3. SNOT-22 and Pulmonary Function

Relative FEV_1_ (%) demonstrated a significant inverse correlation with SNOT-22 total score (r = −0.103, *p* = 0.022; n = 490). Descriptive analysis across SNOT-22 categories showed mean relative FEV_1_ of 86.6% (none), 84.9% (moderate), and 87.5% (severe) (Table 3). Neither absolute FEV_1_ nor vital capacity correlated significantly with SNOT-22 (*p* = 0.171 and *p* = 0.467). The association between SNOT-22 category and obstructive ventilatory pattern (FEV_1_ < 75%) was not significant (*p* = 0.363).

### 3.4. Tobacco Use

Of 500 participants, 57 (11.4%) were smokers. Smokers had numerically higher SNOT-22 mean scores (22.1 vs. 18.3; *p* = 0.092, Mann–Whitney U) and a higher proportion of SNOT-22 ≥ 21 (50.9% vs. 36.6%; *p* = 0.063, Chi^2^); however, neither difference reached statistical significance.

## 4. Discussion

### 4.1. Prevalence of Sinonasal Symptom Burden

The main finding is that 38.2% of young male adults in a population-based setting demonstrated SNOT-22 scores ≥ 21, indicating at least moderate sinonasal symptom burden. This substantially exceeds prior estimates in non-clinical control populations: Farhood et al. reported a mean SNOT-22 of 11.0 (SD 9.4) in 1517 controls [13], and Hopkins et al. reported 9.3 in controls [6]. Normative European data (n = 1000 healthy Germans) established a mean of 20.2 ± 19.4, with females and younger participants scoring higher, underscoring the influence of demographic factors on baseline SNOT-22 values [14]. Females with CRSwNP report significantly higher SNOT-22 scores than males (53.8 vs. 46.8) [15], suggesting that the male-only composition of this cohort may partially explain values below published normative female means despite the high proportion exceeding ≥21.

The elevated prevalence likely reflects several mechanisms. First, this cohort represents a consecutive, unselected sample without known prior ENT consultation, potentially capturing a reservoir of undiagnosed CRS—consistent with US data showing 2.1% of adults meeting CRS criteria without a documented diagnosis [4] and the established radiological–clinical discordance in males [3]. Second, the SNOT-22 captures sleep disturbance and fatigue domains that may be elevated for non-sinonasal reasons in this demographic, potentially increasing scores [13].

Beyond prevalence estimation, these findings suggest that the SNOT-22 could serve as a low-threshold screening instrument to identify undiagnosed sinonasal disease within routine preventive health examinations, with potential downstream benefits for lower airway health given the unified airway concept. However, routine PROM implementation in clinical practice remains inconsistent: a recent Canadian survey found that only half of practising otolaryngologists collect PROM data routinely [16].

### 4.2. CRP, Eosinophils, and Systemic Inflammation

The significant positive correlation between CRP and SNOT-22 (r = 0.117, *p* = 0.009) represents an exploratory finding in a non-clinical population, suggesting a potential association between self-reported sinonasal symptom burden and systemic inflammatory activation. The effect size is modest (r = 0.117, explaining less than 2% of variance), and the result should be interpreted cautiously, particularly given that a large proportion of CRP values were at or near the assay detection floor (median 0.00 mg/dL in the no-symptom group). Concordant findings from a case-control study of 81 CRSwNP patients showed that nasal IL-5 and calprotectin levels were significantly elevated in uncontrolled versus controlled patients and increased with the number of prior sinus surgeries, confirming that local biomarkers mirror systemic disease activity [17]. Nasal or serum cytokine levels (e.g., IL-5) were not measured in the present study; only peripheral blood eosinophil percentage and CRP were available as systemic inflammatory markers, and direct comparison with local cytokine data was therefore not possible.

The monotonic eosinophil gradient across SNOT-22 categories (3.08 → 3.31 → 3.56%) is biologically plausible given the role of eosinophilic inflammation in CRSwNP [10] and the predictive value of blood eosinophilia for biological therapy response [18]. The absence of statistical significance is likely attributable to the low base rate of the Th2-driven eosinophilic CRSwNP endotype in young males, where neutrophilic CRSsNP predominates, and to the small severe subgroup (n = 23). Future studies stratifying by CRS endotype may reveal stronger associations. Similarly, the numeric triglyceride gradient across SNOT-22 categories (87.3 vs. 95.0 vs. 110.2 mg/dL; *p* = 0.059) did not reach significance but is biologically plausible: reciprocal links between blood lipid metabolism and allergic/atopic inflammation, including shared hemostatic and cardiovascular pathways, have been described previously [19], and may partly underlie this exploratory trend.

### 4.3. Pulmonary Function and the Unified Airway

The inverse correlation between SNOT-22 and relative FEV_1_% (r = −0.103, *p* = 0.022) is consistent with the unified airway concept in a healthy young male population, though the effect size is small (less than 2% explained variance) and the descriptive category data showed a non-monotonic pattern (86.6% none, 84.9% moderate, 87.5% severe), likely reflecting the small severe subgroup (n = 23). These findings should therefore be interpreted as exploratory. This is consistent with other studies showing reduced lung function in CRS patients [20] and with prospective evidence that endoscopic sinus surgery reduces new asthma diagnoses [21].

The absence of significant differences in absolute FEV_1_ or vital capacity suggests the relationship is driven by a subtle obstructive pattern detectable only through the relative parameter, not by pre-existing reduced lung volumes—reflecting early or subclinical lower airway involvement. This interpretation is supported by a prospective study demonstrating that eosinophilic nasal polyps correlate with upper–lower airway inflammatory interactions even without comorbid asthma [22], and by strong negative SNOT-22 correlations with pulmonary quality-of-life domains in primary ciliary dyskinesia (r = −0.77) [23].

### 4.4. Strengths and Limitations

Strengths include the large sample size (n = 500), the use of the SNOT-22 in combination with objective laboratory and spirometric measurements, and the population-based non-clinical setting minimising selection bias.

Key limitations are: (1) The cross-sectional design does not allow causal conclusions. (2) The absence of nasal endoscopy and CT imaging prevents confirmation of SNOT-22-positive cases as true CRS per EPOS criteria [24]. A systematic review demonstrated only a moderate correlation between Lund–Mackay CT scores and SNOT-22 (r = 0.43), highlighting the inherent discordance between objective and patient-reported measures [25]. Accordingly, the 38.2% of participants scoring ≥ 21 should be understood as individuals with at least moderate self-reported sinonasal symptom burden, not as a confirmed CRS prevalence estimate. (3) The exclusively male cohort limits generalizability. (4) Allergy status and prior CRS diagnoses were not recorded, and no objective testing for atopic sensitisation (skin prick testing or specific serum IgE) was performed; data on other allergic conditions (asthma, food allergy, atopic dermatitis) were likewise not collected. Undiagnosed allergic rhinitis, which is common in young adults, could independently elevate nasal-domain SNOT-22 scores, potentially inflating the proportion classified as symptomatic and confounding associations with inflammatory markers. (5) The proportion of CRP values at or below the lower limit of assay detection was not formally quantified; a high proportion of floor values may artificially affect Spearman correlations through tied ranks. (6) Tobacco use was captured as a binary self-report (smoker: yes/no); more granular were not collected. (7) Eosinophilic inflammation was assessed only systemically (peripheral blood); nor nasal lavage or FeNO measurement; nasal airway function was not independently assessed: forced oscillometry (small-airway function) and anterior rhinomanometry (nasal obstruction), all were not part of the study protocol and would strengthen future investigation.

## 5. Conclusions

In this population-based cohort of 500 young male adults: (1) 38.2% reported at least moderate sinonasal symptom burden (SNOT-22 ≥ 21); (2) symptom burden correlated weakly but significantly with elevated CRP and reduced relative FEV_1_%, consistent with the unified airway concept, whereas eosinophil gradients remained non-significant; and (3) the SNOT-22 appears feasible as a low-threshold screening tool in preventive health examinations, pending confirmation in prospective, endoscopy/imaging-verified studies including female comparator cohorts.

## Figures and Tables

**Table 1:** SNOT-22 item scores stratified by rhinosinusitis severity (median and IQR).

SNOT-22 Item	Total			Rhinosinusitis Score								
				None (0–20)			Moderate (21–50)			Severe (>50)		
	Median	Q1	Q3	Median	Q1	Q3	Median	Q1	Q3	Median	Q1	Q3
2.1 Need to blow nose	1.00	0.00	2.00	1.00	0.00	1.00	2.00	1.00	3.00	3.00	2.00	4.00
2.2 Nasal blockage/obstruction	1.00	0.00	2.00	1.00	0.00	1.00	2.00	1.00	3.00	3.00	3.00	4.00
2.3 Sneezing	1.00	0.00	2.00	0.00	0.00	1.00	1.00	1.00	2.00	2.00	1.00	3.00
2.4 Runny nose	1.00	0.00	2.00	0.00	0.00	1.00	2.00	1.00	3.00	3.00	2.00	4.00
2.5 Cough	1.00	0.00	2.00	0.00	0.00	1.00	2.00	1.00	3.00	3.00	2.00	4.00
2.6 Post-nasal discharge	1.00	0.00	2.00	0.00	0.00	1.00	1.00	0.00	3.00	3.00	3.00	4.00
2.7 Thick nasal discharge	0.00	0.00	1.00	0.00	0.00	0.00	1.00	0.00	2.00	3.00	2.00	3.00
2.11 Facial pain/pressure	0.00	0.00	0.00	0.00	0.00	0.00	0.00	0.00	1.00	1.00	0.00	3.00
2.12 Reduced sense of smell/taste	0.00	0.00	1.00	0.00	0.00	0.00	0.00	0.00	1.00	2.00	1.00	3.00
*SNOT-22 Subscale 1—Nasal Symptoms (items 1–7, 11, 12)*	0.78	0.33	1.44	0.44	0.22	0.78	1.44	1.06	2.00	2.44	2.11	2.89
2.8 Ear fullness	0.00	0.00	1.00	0.00	0.00	0.00	1.00	0.00	2.00	3.00	1.00	4.00
2.9 Dizziness	0.00	0.00	1.00	0.00	0.00	0.00	1.00	0.00	1.00	2.00	1.00	3.00
2.10 Ear pain	0.00	0.00	0.00	0.00	0.00	0.00	0.00	0.00	1.00	3.00	0.00	4.00
*SNOT-22 Subscale 2—Ear/Facial Symptoms (items 8–10)*	0.33	0.00	0.67	0.00	0.00	0.33	0.67	0.33	1.33	2.67	1.67	3.33
2.13 Difficulty falling asleep	0.00	0.00	2.00	0.00	0.00	1.00	1.00	0.00	3.00	3.00	1.00	5.00
2.14 Waking up at night	0.00	0.00	1.00	0.00	0.00	0.00	1.00	0.00	3.00	3.00	1.00	4.00
2.15 Lack of good night’s sleep	0.00	0.00	2.00	0.00	0.00	0.00	2.00	1.00	3.00	4.00	2.00	4.00
2.16 Waking up tired	2.00	0.00	3.00	1.00	0.00	2.00	3.00	2.00	4.00	4.00	3.00	5.00
2.17 Fatigue	1.00	0.00	2.00	0.00	0.00	1.00	2.00	1.00	3.00	4.00	3.00	4.00
*SNOT-22 Subscale 3—Sleep Symptoms (items 13–17)*	0.80	0.20	1.80	0.40	0.00	0.80	1.80	1.20	2.60	3.40	2.40	4.00
2.18 Reduced productivity	0.00	0.00	1.00	0.00	0.00	0.00	1.00	1.00	2.00	3.00	2.00	4.00
2.19 Reduced concentration	0.00	0.00	1.00	0.00	0.00	0.00	1.00	1.00	2.00	4.00	2.00	4.00
2.20 Frustrated/restless/irritable	0.00	0.00	1.00	0.00	0.00	0.00	1.00	0.00	2.00	3.00	1.00	4.00
2.21 Sad	0.00	0.00	1.00	0.00	0.00	0.00	0.00	0.00	1.00	2.00	0.00	3.00
2.22 Embarrassed by sinus symptoms	0.00	0.00	0.00	0.00	0.00	0.00	0.00	0.00	0.00	2.00	0.00	3.00
*SNOT-22 Subscale 4—Emotional Symptoms (items 18–22)*	0.40	0.00	1.00	0.00	0.00	0.40	1.00	0.60	1.60	2.60	2.00	3.20
SNOT-22 Total Score	15.00	7.00	27.00	9.00	4.00	14.00	30.00	24.00	37.00	58.00	54.00	63.00

IQR = interquartile range; Q1 = 25th percentile; Q3 = 75th percentile. Rhinosinusitis severity classified by visual analogue scale (VAS): none 0–20, moderate 21–50, severe >50.

**Table 2:** Spearman correlations between SNOT-22 total score and selected continuous parameters (n = 500).

Variable	Spearman r	*p*-Value	n
CRP (mg/dL)	0.117	0.009 *	500
Relative FEV_1_ (% of VC)	−0.103	0.022 *	490
Erythrocytes (TPt/L)	0.091	0.042 *	500
Triglycerides (mg/dL)	0.084	0.059	500
Eosinophils (%)	0.063	0.159	500
BMI (kg/m^2^)	0.058	0.198	500
Waist circumference (cm)	0.073	0.101	500
Cholesterol (mg/dL)	0.078	0.083	500

* *p* < 0.05 (two-tailed).

**Table 3:** Spirometric parameters across SNOT-22 categories.

Parameter	n Valid	SNOT 0–20 (None)	SNOT 21–50 (mod.)	SNOT > 50 (Severe)
Relative FEV_1_%, mean (SD)	490	86.6 (6.5)	84.9 (6.1)	87.5 (4.9)
FEV_1_ (l), mean (SD)	499	4.72 (0.63)	4.70 (0.61)	4.60 (0.45)
Vital capacity (l), mean (SD)	500	5.46 (0.72)	5.49 (0.74)	5.26 (0.44)
FEV_1_ < 75%, n (%)	490	16 (5.2%)	11 (6.8%)	0 (0.0%)

SD = standard deviation; FEV_1_ = forced expiratory volume in 1 s; mod. = moderate.

## Data Availability

The data presented in this study are available on reasonable request from the corresponding author. The data are not publicly available due to privacy restrictions.

## Abbreviations

The following abbreviations are used in this manuscript:

BMI	body mass index
CRP	C-reactive protein
CRS	chronic rhinosinusitis
CRSsNP	chronic rhinosinusitis without nasal polyps
CRSwNP	chronic rhinosinusitis with nasal polyps
CT	computed tomography
EPOS	European Position Paper on Rhinosinusitis and Nasal Polyps
FEV_1_	forced expiratory volume in 1 s
IQR	interquartile range
PROM	patient-reported outcome measure
SNOT-22	22-item Sinonasal Outcome Test
VAS	visual analogue scale
VC	vital capacity
